
JOURNAL INFORMATION
==============================
NLM Title Abbreviation: Can Med Educ J
Journal ID: Can Med Educ J
NLM Journal ID: 101560935
Journal ID: CMEJ

Canadian Medical Education Journal

EISSN: 1923-1202
Publisher: University of Saskatchewan

ARTICLE INFORMATION
==============================
PMCID: PMC6448506
Article ID: CMEJ-10-e003
Article version: 1
Subjects: Abstracts

Workshop Abstracts

Electronic publication date: 2019 Apr 3
Publication date: 2019 Apr
Volume: 10
Issue: 2
First page: e003
Last page: e028
Copyright year: 2019
License: This is an Open Journal Systems article distributed under the terms of the Creative Commons Attribution License (http://creativecommons.org/licenses/by/2.0) which permits unrestricted use, distribution, and reproduction in any medium, provided the original work is properly cited.
License URL: https://creativecommons.org/licenses/by/2.0/

==============================
JOURNAL INFORMATION
==============================
NLM Title Abbreviation: Can Med Educ J
Journal ID: Can Med Educ J
NLM Journal ID: 101560935
Journal ID: CMEJ

Canadian Medical Education Journal

EISSN: 1923-1202
Publisher: University of Saskatchewan

ARTICLE INFORMATION
==============================
Subjects: Wa - 1

CPD eCoach: An online, self-guided practice assessment tool to support individual learning and practice improvement

Lynn Brenna University of British Columbia

Bluman Bob University of British Columbia

Newton Christie University of British Columbia

Kirshin Toby BC College of Family Physicians

Beamish Laura University of British Columbia

Lam Vivian University of British Columbia

Hobson Bruce University of British Columbia

Barrows Jennie University of British Columbia

Electronic publication date: 2019 Apr 3

JOURNAL INFORMATION
==============================
NLM Title Abbreviation: Can Med Educ J
Journal ID: Can Med Educ J
NLM Journal ID: 101560935
Journal ID: CMEJ

Canadian Medical Education Journal

EISSN: 1923-1202
Publisher: University of Saskatchewan

ARTICLE INFORMATION
==============================
Subjects: Wa - 2

The art of facilitation: Learning to facilitate learning

Penciner Rick University of Toronto

Rezmovitz Jeremy University of Toronto

Electronic publication date: 2019 Apr 3

JOURNAL INFORMATION
==============================
NLM Title Abbreviation: Can Med Educ J
Journal ID: Can Med Educ J
NLM Journal ID: 101560935
Journal ID: CMEJ

Canadian Medical Education Journal

EISSN: 1923-1202
Publisher: University of Saskatchewan

ARTICLE INFORMATION
==============================
Subjects: Wa - 3

Preceptor-learner boundaries: Optimizing professional, educational and personal relationships

Goertzen James Northern Ontario School of Medicine

Boillat Miriam McGill

Cavett Teresa University of Manitoba

Kvern Brent University of Manitoba

Electronic publication date: 2019 Apr 3

JOURNAL INFORMATION
==============================
NLM Title Abbreviation: Can Med Educ J
Journal ID: Can Med Educ J
NLM Journal ID: 101560935
Journal ID: CMEJ

Canadian Medical Education Journal

EISSN: 1923-1202
Publisher: University of Saskatchewan

ARTICLE INFORMATION
==============================
Subjects: Wa - 4

Effective teaching practices that support learner (and teacher) mental health and wellbeing

D'Eon Marcel University of Saskatchewan

Polreis Sean University of Saskatchewan

MacLean Cathy University of Saskatchewan

Chang Helen University of Saskatchewan

McKague Meredith University of Saskatchewan

Epstein Michael University of Saskatchewan

Electronic publication date: 2019 Apr 3

JOURNAL INFORMATION
==============================
NLM Title Abbreviation: Can Med Educ J
Journal ID: Can Med Educ J
NLM Journal ID: 101560935
Journal ID: CMEJ

Canadian Medical Education Journal

EISSN: 1923-1202
Publisher: University of Saskatchewan

ARTICLE INFORMATION
==============================
Subjects: Wa - 5

Don't Fail to Fail: Strategies for Clear Communication with the Learner in Difficulty

Wiebe Carmen University of Toronto

Halman Mark University of Toronto

Electronic publication date: 2019 Apr 3

JOURNAL INFORMATION
==============================
NLM Title Abbreviation: Can Med Educ J
Journal ID: Can Med Educ J
NLM Journal ID: 101560935
Journal ID: CMEJ

Canadian Medical Education Journal

EISSN: 1923-1202
Publisher: University of Saskatchewan

ARTICLE INFORMATION
==============================
Subjects: Wa - 6

The Challenges of High Quality Qualitative Research

Nimmon Laura University of British Columbia

Watling Christopher Western University

Baker Lindsay University of Toronto

Nimmon Laura University of British Columbia

Watling Christopher Western University

Electronic publication date: 2019 Apr 3

JOURNAL INFORMATION
==============================
NLM Title Abbreviation: Can Med Educ J
Journal ID: Can Med Educ J
NLM Journal ID: 101560935
Journal ID: CMEJ

Canadian Medical Education Journal

EISSN: 1923-1202
Publisher: University of Saskatchewan

ARTICLE INFORMATION
==============================
Subjects: Wb - 1

Writing reliable Multiple Choice Questions (MCQ)s, and using item analysis for post-exam analysis

Martiin Susanna University of Saskatchewan

Lloyd Joshua University of Saskatchewan

Electronic publication date: 2019 Apr 3

JOURNAL INFORMATION
==============================
NLM Title Abbreviation: Can Med Educ J
Journal ID: Can Med Educ J
NLM Journal ID: 101560935
Journal ID: CMEJ

Canadian Medical Education Journal

EISSN: 1923-1202
Publisher: University of Saskatchewan

ARTICLE INFORMATION
==============================
Subjects: Wb - 2

So you want to Implement Programmatic Assessment? Strategies, tactics, and lessons learned from the University of British Columbia (UBC) and the University of Toronto Experiences.

Tait Glendon University of Toronto

Veerapen Kiran University of British Columbia

Electronic publication date: 2019 Apr 3

JOURNAL INFORMATION
==============================
NLM Title Abbreviation: Can Med Educ J
Journal ID: Can Med Educ J
NLM Journal ID: 101560935
Journal ID: CMEJ

Canadian Medical Education Journal

EISSN: 1923-1202
Publisher: University of Saskatchewan

ARTICLE INFORMATION
==============================
Subjects: Wb - 3

Managing Change in Medical Education

Damon Dagnone J. Queen’s University

Chan Ming-Ka University of Manitoba

Meschino Diane University of Toronto

McEwen Laura Queen’s University

Electronic publication date: 2019 Apr 3

JOURNAL INFORMATION
==============================
NLM Title Abbreviation: Can Med Educ J
Journal ID: Can Med Educ J
NLM Journal ID: 101560935
Journal ID: CMEJ

Canadian Medical Education Journal

EISSN: 1923-1202
Publisher: University of Saskatchewan

ARTICLE INFORMATION
==============================
Subjects: Wb - 4

Art is Patient With Us: proposal for using video art in place of live patient narrative

Matamoros Cam University of Calgary

Rosenal Tom University of Calgary

Surkan Neil University of Calgary

Electronic publication date: 2019 Apr 3

JOURNAL INFORMATION
==============================
NLM Title Abbreviation: Can Med Educ J
Journal ID: Can Med Educ J
NLM Journal ID: 101560935
Journal ID: CMEJ

Canadian Medical Education Journal

EISSN: 1923-1202
Publisher: University of Saskatchewan

ARTICLE INFORMATION
==============================
Subjects: Wb - 5

Disrupting our teaching practices: Applying self-determination theory to set the motivational context for learning

Malin Greg University of Saskatchewan

Electronic publication date: 2019 Apr 3

JOURNAL INFORMATION
==============================
NLM Title Abbreviation: Can Med Educ J
Journal ID: Can Med Educ J
NLM Journal ID: 101560935
Journal ID: CMEJ

Canadian Medical Education Journal

EISSN: 1923-1202
Publisher: University of Saskatchewan

ARTICLE INFORMATION
==============================
Subjects: Wb - 6

Understanding and utilizing item analysis to create high-quality multiple-choice questions.

Lloyd Joshua University of Saskatchewan

Martin Susanna University of Saskatchewan

Electronic publication date: 2019 Apr 3

JOURNAL INFORMATION
==============================
NLM Title Abbreviation: Can Med Educ J
Journal ID: Can Med Educ J
NLM Journal ID: 101560935
Journal ID: CMEJ

Canadian Medical Education Journal

EISSN: 1923-1202
Publisher: University of Saskatchewan

ARTICLE INFORMATION
==============================
Subjects: Wc - 1

Speaking From The Heart: Addressing The Language Learning Needs of Multilingual Medical Students

Narayan Lalit George Washington School of Medicine and Health Sciences

Tran Wynn University of British Columbia

Zhuang Meiying University of British Columbia

Costa Vonessa Cambridge Health Alliance,

Electronic publication date: 2019 Apr 3

JOURNAL INFORMATION
==============================
NLM Title Abbreviation: Can Med Educ J
Journal ID: Can Med Educ J
NLM Journal ID: 101560935
Journal ID: CMEJ

Canadian Medical Education Journal

EISSN: 1923-1202
Publisher: University of Saskatchewan

ARTICLE INFORMATION
==============================
Subjects: Wc - 2

Power dynamics in the learning and work environment: 'watt' can we do?

Chan Ming-Ka University of Manitoba

Dath Deepak McMaster University

de Camps Meschino Diane University of Toronto

Electronic publication date: 2019 Apr 3

JOURNAL INFORMATION
==============================
NLM Title Abbreviation: Can Med Educ J
Journal ID: Can Med Educ J
NLM Journal ID: 101560935
Journal ID: CMEJ

Canadian Medical Education Journal

EISSN: 1923-1202
Publisher: University of Saskatchewan

ARTICLE INFORMATION
==============================
Subjects: Wc - 3

Learning in an Artificial Intelligence World

Peteanu Wanda The Michener Institute, University Health Network

Gillan Caitlin University of Toronto

Salhia Mohammad The Michener Institute, University Health Network

Wiljer David University of Toronto

Electronic publication date: 2019 Apr 3

JOURNAL INFORMATION
==============================
NLM Title Abbreviation: Can Med Educ J
Journal ID: Can Med Educ J
NLM Journal ID: 101560935
Journal ID: CMEJ

Canadian Medical Education Journal

EISSN: 1923-1202
Publisher: University of Saskatchewan

ARTICLE INFORMATION
==============================
Subjects: Wc - 4

Doctors Against Tragedies - Fighting the Fentanyl Crisis through Game Play

Maruyama Michiko University of Alberta

Mack Cheryl University of Alberta

Delmar Lindsay University of Alberta

Arunachalam Meyy University of Alberta

Szerb Jennifer Dalhousie University

Electronic publication date: 2019 Apr 3

JOURNAL INFORMATION
==============================
NLM Title Abbreviation: Can Med Educ J
Journal ID: Can Med Educ J
NLM Journal ID: 101560935
Journal ID: CMEJ

Canadian Medical Education Journal

EISSN: 1923-1202
Publisher: University of Saskatchewan

ARTICLE INFORMATION
==============================
Subjects: Wc - 5

When theory hits the real world: Exploring tensions around entrustment in non-procedural clinical contexts

Hatala Rose University of British Columbia

Gingerich Andrea University of British Columbia

Ginsburg Shiphra University of Toronto

Goldszmidt Mark Western University

Electronic publication date: 2019 Apr 3

JOURNAL INFORMATION
==============================
NLM Title Abbreviation: Can Med Educ J
Journal ID: Can Med Educ J
NLM Journal ID: 101560935
Journal ID: CMEJ

Canadian Medical Education Journal

EISSN: 1923-1202
Publisher: University of Saskatchewan

ARTICLE INFORMATION
==============================
Subjects: Wc - 6

Adjusting to new contexts of clinical training

Bates Joanna University of British Columbia

Watling Christopher Western University

Schrewe Brett University of British Columbia

Ellaway Rachel University of Calgary

Teunissen Pim University of Maastricht

Electronic publication date: 2019 Apr 3

JOURNAL INFORMATION
==============================
NLM Title Abbreviation: Can Med Educ J
Journal ID: Can Med Educ J
NLM Journal ID: 101560935
Journal ID: CMEJ

Canadian Medical Education Journal

EISSN: 1923-1202
Publisher: University of Saskatchewan

ARTICLE INFORMATION
==============================
Subjects: Wd - 1

How to "AACE-IT:" Coaching Students in Difficulty - Lessons learned from the Achieving Academic and Clinical Excellence In Training Program at the University of Toronto

Premji Laila University of Toronto

Tzanetos Katina University of Toronto

Lazor Jana University of Toronto

Electronic publication date: 2019 Apr 3

JOURNAL INFORMATION
==============================
NLM Title Abbreviation: Can Med Educ J
Journal ID: Can Med Educ J
NLM Journal ID: 101560935
Journal ID: CMEJ

Canadian Medical Education Journal

EISSN: 1923-1202
Publisher: University of Saskatchewan

ARTICLE INFORMATION
==============================
Subjects: Wd - 2

Developing Partnerships between Community Organizations and Universities to Deliver Service Learning Experiences for Medical Students

Cook Karen University of Manitoba

Wright Roxanne University of Toronto

Jalloh Chelsea University of Manitoba

Peddle Sarah Dalhousie University

Cook Karen University of Manitoba

Andermann Anne McGill

Whetter Ian University of Manitoba

Carlin Robert McGill

Electronic publication date: 2019 Apr 3

JOURNAL INFORMATION
==============================
NLM Title Abbreviation: Can Med Educ J
Journal ID: Can Med Educ J
NLM Journal ID: 101560935
Journal ID: CMEJ

Canadian Medical Education Journal

EISSN: 1923-1202
Publisher: University of Saskatchewan

ARTICLE INFORMATION
==============================
Subjects: Wd - 3

Competence Committees: Designing and Running an Effective Competence Committee to Support Resident Progress

Johnstone Julie University of Toronto

Atkinson Adelle University of Toronto

Electronic publication date: 2019 Apr 3

JOURNAL INFORMATION
==============================
NLM Title Abbreviation: Can Med Educ J
Journal ID: Can Med Educ J
NLM Journal ID: 101560935
Journal ID: CMEJ

Canadian Medical Education Journal

EISSN: 1923-1202
Publisher: University of Saskatchewan

ARTICLE INFORMATION
==============================
Subjects: Wd - 4

Using DELPHI Methodologies in Medical Education Research - the Basics of Design and Deployment

Murray John PhD University of Manitoba

Alexiadis-Brown MA Peggy Dalhousie University

Electronic publication date: 2019 Apr 3

JOURNAL INFORMATION
==============================
NLM Title Abbreviation: Can Med Educ J
Journal ID: Can Med Educ J
NLM Journal ID: 101560935
Journal ID: CMEJ

Canadian Medical Education Journal

EISSN: 1923-1202
Publisher: University of Saskatchewan

ARTICLE INFORMATION
==============================
Subjects: Wd - 5

Teaching with Serious Board Games: Learning from the GridlockED Example

Chan Teresa McMaster University

Bhalerao Anuja University of Toronto

Suryavanshi Tanishq McMaster University

Electronic publication date: 2019 Apr 3

JOURNAL INFORMATION
==============================
NLM Title Abbreviation: Can Med Educ J
Journal ID: Can Med Educ J
NLM Journal ID: 101560935
Journal ID: CMEJ

Canadian Medical Education Journal

EISSN: 1923-1202
Publisher: University of Saskatchewan

ARTICLE INFORMATION
==============================
Subjects: Wd - 6

Understanding the Role of Faculty Teachers and Educators in CBME : Coaching and Competency

Antao Viola University of Toronto

Leslie Karen University of Toronto

Electronic publication date: 2019 Apr 3

JOURNAL INFORMATION
==============================
NLM Title Abbreviation: Can Med Educ J
Journal ID: Can Med Educ J
NLM Journal ID: 101560935
Journal ID: CMEJ

Canadian Medical Education Journal

EISSN: 1923-1202
Publisher: University of Saskatchewan

ARTICLE INFORMATION
==============================
Subjects: We - 1

Curriculum Mapping to Broaden Scope and Impact of Your Continuing Professional Development Program Development

Lochnan Heather University of Ottawa

Parson Robert University of Ottawa

Hendry Paul University of Ottawa

Electronic publication date: 2019 Apr 3

JOURNAL INFORMATION
==============================
NLM Title Abbreviation: Can Med Educ J
Journal ID: Can Med Educ J
NLM Journal ID: 101560935
Journal ID: CMEJ

Canadian Medical Education Journal

EISSN: 1923-1202
Publisher: University of Saskatchewan

ARTICLE INFORMATION
==============================
Subjects: We - 2

"Falling Through the Cracks": A Patient-Centered Film Creates Meaning for an Undergraduate Teamwork Curriculum

Fraser Kristin University of Calgary

Flemons Ward University of Calgary

Price Teri Greg's Wings

Charania Irina University of Calgary

Sharma Nishan University of Calgary

Wishart Ian University of Calgary

Electronic publication date: 2019 Apr 3

JOURNAL INFORMATION
==============================
NLM Title Abbreviation: Can Med Educ J
Journal ID: Can Med Educ J
NLM Journal ID: 101560935
Journal ID: CMEJ

Canadian Medical Education Journal

EISSN: 1923-1202
Publisher: University of Saskatchewan

ARTICLE INFORMATION
==============================
Subjects: We - 3

Adapting to Change: Assessment and Coaching in Portfolio

Bernhard Nirit University of Toronto

Talarico Susanna University of Toronto

Talarico Susanna University of Toronto

Tait Glendon University of Toronto

Bryden Pier University of Toronto

Sargeant Joan Dalhousie University

Electronic publication date: 2019 Apr 3

JOURNAL INFORMATION
==============================
NLM Title Abbreviation: Can Med Educ J
Journal ID: Can Med Educ J
NLM Journal ID: 101560935
Journal ID: CMEJ

Canadian Medical Education Journal

EISSN: 1923-1202
Publisher: University of Saskatchewan

ARTICLE INFORMATION
==============================
Subjects: We - 4

Live One, Teach One: Building equitable collaborations with health service users to transform health professions education

Agrawal Sacha University of Toronto

Beder Michaela University of Toronto

Berkhout Suze University of Toronto

Cooper Rachel University of Toronto

Kalocsai Csilla Centre for Addiction and Mental Health

McGovern Brenda University of Toronto

Soklaridis Sophie University of Toronto

Wiljer David University of Toronto

Electronic publication date: 2019 Apr 3

JOURNAL INFORMATION
==============================
NLM Title Abbreviation: Can Med Educ J
Journal ID: Can Med Educ J
NLM Journal ID: 101560935
Journal ID: CMEJ

Canadian Medical Education Journal

EISSN: 1923-1202
Publisher: University of Saskatchewan

ARTICLE INFORMATION
==============================
Subjects: We - 5

Utilizing Design Thinking to Guide Design and Development of Online Patient and Healthcare Professional Education

Bayer Ilana McMaster University

Leyland Margaret McMaster University

Electronic publication date: 2019 Apr 3

JOURNAL INFORMATION
==============================
NLM Title Abbreviation: Can Med Educ J
Journal ID: Can Med Educ J
NLM Journal ID: 101560935
Journal ID: CMEJ

Canadian Medical Education Journal

EISSN: 1923-1202
Publisher: University of Saskatchewan

ARTICLE INFORMATION
==============================
Subjects: We - 6

Designing Curricula to Maximize Learning

Stewart Wendy Dalhousie University

Wilson Keith Dalhousie University

Electronic publication date: 2019 Apr 3

JOURNAL INFORMATION
==============================
NLM Title Abbreviation: Can Med Educ J
Journal ID: Can Med Educ J
NLM Journal ID: 101560935
Journal ID: CMEJ

Canadian Medical Education Journal

EISSN: 1923-1202
Publisher: University of Saskatchewan

ARTICLE INFORMATION
==============================
Subjects: Wf - 1

Learning from the 'Kolabo' experience: Navigating challenges and alternative approaches to small group learning in low resource settings

Arcand Jaylynn University of Calgary

Grimminck Rachel University of Calgary

Williams Kimberly University of Calgary

Li Jordan University of Calgary

Fitzgerald Critstin University of Calgary

Watterson Rita University of Calgary

Poon Susan University of Calgary

Grimminck Michael University of Calgary

Mohan Kanwal University of Calgary

Choo Jian University of Calgary

Dornian Jonathon University of Calgary

Shen Mary University of Calgary

Mwita Matiko
Electronic publication date: 2019 Apr 3

JOURNAL INFORMATION
==============================
NLM Title Abbreviation: Can Med Educ J
Journal ID: Can Med Educ J
NLM Journal ID: 101560935
Journal ID: CMEJ

Canadian Medical Education Journal

EISSN: 1923-1202
Publisher: University of Saskatchewan

ARTICLE INFORMATION
==============================
Subjects: Wf - 3

Medical Learner Mistreatment: Exploring Directions and Strategies for Enhancing Success of Mistreatment Interventions

Gupta Namta McGill

Velez Camila McGill

Electronic publication date: 2019 Apr 3

JOURNAL INFORMATION
==============================
NLM Title Abbreviation: Can Med Educ J
Journal ID: Can Med Educ J
NLM Journal ID: 101560935
Journal ID: CMEJ

Canadian Medical Education Journal

EISSN: 1923-1202
Publisher: University of Saskatchewan

ARTICLE INFORMATION
==============================
Subjects: Wf - 4

Resident Leadership in the Co-Production of CBME

Buttemer Samantha Queen’s University

Weersink Kristen Queen’s University

Hall Jena Queen’s University

Dagnone Damon Queen’s University

Tai Julia Queen’s University

Hay Kathryn Queen’s University

Berger Liora Queen’s University

Trier Jessica Queen’s University

Electronic publication date: 2019 Apr 3

JOURNAL INFORMATION
==============================
NLM Title Abbreviation: Can Med Educ J
Journal ID: Can Med Educ J
NLM Journal ID: 101560935
Journal ID: CMEJ

Canadian Medical Education Journal

EISSN: 1923-1202
Publisher: University of Saskatchewan

ARTICLE INFORMATION
==============================
Subjects: Wf - 5

How can we deliver learner-driven mentoring in an academic setting?

Ameyaw Stephanie University of British Columbia

Lynn Brenna University of British Columbia

Parhar Gurdeep University of British Columbia

Bluman Bob University of British Columbia

Paul Susan University of British Columbia

Electronic publication date: 2019 Apr 3

JOURNAL INFORMATION
==============================
NLM Title Abbreviation: Can Med Educ J
Journal ID: Can Med Educ J
NLM Journal ID: 101560935
Journal ID: CMEJ

Canadian Medical Education Journal

EISSN: 1923-1202
Publisher: University of Saskatchewan

ARTICLE INFORMATION
==============================
Subjects: Wf - 6

An interprofessional education course on design thinking for technology

Tang Sabrina Dalhousie University

Braunizer Anna Vancouver Coastal Health

Miller Stephen Dr. Dalhousie University

Lauckner Heidi Professor Dalhousie University

Electronic publication date: 2019 Apr 3

JOURNAL INFORMATION
==============================
NLM Title Abbreviation: Can Med Educ J
Journal ID: Can Med Educ J
NLM Journal ID: 101560935
Journal ID: CMEJ

Canadian Medical Education Journal

EISSN: 1923-1202
Publisher: University of Saskatchewan

ARTICLE INFORMATION
==============================
Subjects: Wf - 7

Best Practices in Medicine (BPiM) - How to Organize and Implement a Right-Sizing Test Utilization Campaign

Jackman Mallory University of Toronto

Kapur Ajay University of Toronto

Seth Rishie University of Toronto

Tron Victor University of Toronto

Kherani Raheem University of British Columbia

Barnes Craig St Joseph's Health Centre

Arab-O'Brien Donna University of Toronto

Maniate Jerry University of Ottawa

Mulsant Sharon University of Toronto

Pasic Maria University of Toronto

Taher Jennifer University of Toronto

Wooster Elizabeth OISE/University of Toronto

Electronic publication date: 2019 Apr 3

